# Design and Applications of Polymersomes for Oral Drug Administration

**DOI:** 10.1021/acsami.5c04658

**Published:** 2025-05-15

**Authors:** Wing-Fu Lai

**Affiliations:** School of Food Science and Nutrition, 4468 University of Leeds, Leeds LS2 9JT, United Kingdom

**Keywords:** polymersomes, block copolymers, colloidal behavior, drug delivery, oral administration, controlled release, amphiphilicity

## Abstract

Polymersomes are nanostructures consisting of a hollow aqueous compartment enclosed by a coating of amphiphilic block copolymers. Owing to the entangled nature of their membrane, polymersomes exhibit higher mechanical stability than some other extensively studied nanostructures such as liposomes. This also enables the properties of the polymersome membrane to be more easily tuned to meet practical needs, making polymersomes promising carriers for drug delivery. Since the turn of the last century, the use of polymersomes has been exploited in diverse areas, ranging from protein therapy to medical imaging. Yet, discussions exploring the opportunities and challenges of the development of polymersomes for oral drug administration have been scant. This review addresses this gap by offering a snapshot of the current advances in the design, fabrication, and use of polymersomes as oral drug carriers. It is hoped that this review will not only highlight the practical potential of polymersomes for oral drug administration but will also shed light on the challenges determining the wider clinical potential of polymersomes in the forthcoming decades.

## Graphical abstract

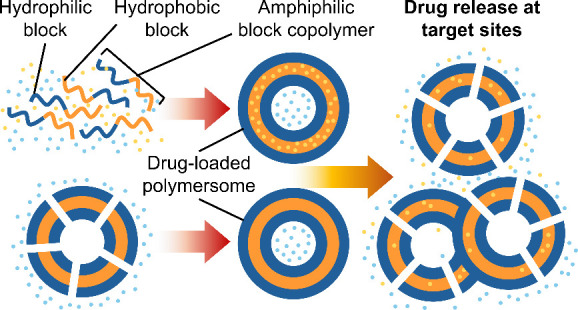

## Introduction

Polymersomes (also known as polymeric vesicles) are nanostructures consisting of a hallow aqueous compartment enclosed by a coating of amphiphilic block copolymers that undergo self-assembly during polymersome fabrication.^1–3^ Compared with homopolymers and various types of copolymers (including random copolymers and alternate copolymers), block copolymers show unique tunability in structures and physical properties.^4^ This makes fine-tuning of the colloidal behavior via changes in the chain length and in the structure of the block segment feasible. Owing to this feature, amphiphilic block copolymers are known to be able to form diverse types of particulate vehicles, ranging from worm-like micelles to polymersomes in an aqueous environment.^5–7^ Over the years, various block copolymers [such as poly­(ethylene glycol)-*b*-poly­(amino acid),^8^ poly­(ethylene glycol)-*b*-poly­(ε-caprolactone),^9^ and poly­(ethylene glycol)-*b*-poly­(*D*,*L*-lactide)^10^] have demonstrated the capacity of forming micelles via self-assembly. The generated micelles have been successfully adopted to deliver therapeutic agents. Recently, polymersomes generated by a folate-conjugated Pluronic P85/poly­(lactide-*co*-glycolide) (FA-P85-PLGA) copolymer have been exploited for insulin delivery to fasting diabetic rats.^11^ While no hypoglycemic effect has been observed in the group administered with free insulin, rats administered with insulin-loaded polymersomes have exhibited significant and prolonged hypoglycemic effects.^11^ This corroborates the clinical potential of polymersomes in pharmaceutical formulation.

Compared with many other carrier systems (e.g., liposomes, micelles, and solid lipid nanoparticles), polymersomes show distinct advantages for mediating oral drug delivery. Their unique vesicular architecture, formed by the self-assembly of amphiphilic block copolymers, results in a bilayer membrane that is significantly thicker and more stable than that of liposomes.^12^ This enhanced membrane robustness offers superior protection for encapsulated drugs. Additionally, the physicochemical properties of polymersomes—such as size, surface charge, membrane permeability, and degradation rate—can be finely tuned through precise control of the polymer composition and architecture.^13^ This tunability enhances the ability of polymersomes to overcome the physiological and biochemical barriers associated with drug administration. Unlike many micellar systems, polymersomes are less prone to premature disassembly due to their kinetic stability.^14^ This facilitates sustained drug release. Combined with their capacity to encapsulate both hydrophilic and hydrophobic drugs and their ease of surface functionalization,^15,16^ these features position polymersomes as a versatile and highly customizable platform for drug delivery.

Up to now, the use of polymersome-based carriers has already been exploited in diverse areas, ranging from protein therapy^17,18^ to medical imaging.^19–21^ Despite this, most of the studies in the literature have exploited polymersomes mainly as carriers for systemic drug administration.^22–26^ Efforts devoted to exploring the potential use of polymersomes as oral drug carriers have been scant. In fact, compared to parenteral routes (e.g., intravenous, subcutaneous, and intramuscular routes), drug administration via the oral route has unique advantages ranging from noninvasiveness and convenience of operation to high patient compliance. Approximately 60% of commercially available small-molecule pharmaceutical products are administered via the oral route,^27^ with around 90% of the global market share of all drug formulations intended for human use being estimated to be taken up by oral formulations.^27^ Due to the presence of multiple barriers—ranging from the harsh gastric environment to metabolic breakdown of the drug in the intestinal region—unique to oral drug administration, achieving high efficiency of drug delivery via the oral route is more challenging than via other parenteral methods (Table 1).^28,29^ The objective of this article is to revisit the role of polymersomes in oral drug delivery by reviewing the latest advances in the design, fabrication, and optimization of polymersomes as oral drug carriers.

**Table 1:** Barriers Imposed by Different Parts of the Gastrointestinal Tract for Polymersome-Mediated Oral Drug Administration

region	pH	transit time	features	ref
oral cavity	6.8–7.0	0.4–13 s	high accessibility for drug administration	^65–68^
			limited surface area for drug absorption	
			presence of saliva and enzymes as barriers of drug delivery	
esophagus	6.8–7.0	1–8 s	short residence time of the administered agent for proper absorption	
			low permeability to drug molecules	
stomach	1.2–2.0	3–4 h	provision of a highly acidic environment that inactivates the administered agent	
			presence of tight junctions to limit drug absorption	
			presence of pepsins to inactivate proteinaceous drugs	
small intestine	6.0–7.4	2–6 h	provision of a large surface area for drug adsorption	
			action of pancreatic enzymes and bile salts, along with the presence of the mucosal layer in the lining of the intestinal tract, reduces oral bioavailability of the administered agent	
			elimination of the administered agent by intestinal metabolism triggered by digestive enzymes	
			brush-border metabolism of the administered agent mediated by the digestive enzymes present in the brush border of microvilli	
			intracellular metabolism of the drug molecules in the enterocytes under the action of various enzymes (including cytochrome P450 enzymes and phase II conjugating enzymes)	
large intestine and rectum	6.0–6.7	6–70 h	lower extent of enzymatic activity compared to other parts of the gastrointestinal tract	
			longer residence time of the administered agent	
			metabolism of the administered agent mediated by the gut microflora	

## Structural Design of Block Copolymers and the Polymersome Thereof

Structures of block copolymers play a vital role in determining the properties (including but not limited to physical stability and membrane thickness) of the polymersomes generated. Such properties, in turn, affect the drug encapsulation efficiency, drug release sustainability, and metabolic fate of the polymersomes upon oral ingestion. To render the copolymers amphiphilic, both hydrophilic and hydrophobic blocks must be incorporated into their structures. Poly­(acrylate), poly­(lactic acid), poly­(caprolactone) (PCL), and poly­(methacrylate) are some of the commonly used candidates for the hydrophobic block, although other polymers such as polydimethylsiloxane, poly­(γ-benzyl-l-glutamate), polystyrene, poly­(trymethylene carbonate), and poly­(2-oxazoline) have been adopted in the literature.^30^ For the hydrophilic block, poly­(acrylic acid) (PAA), polyacrylamides, poly­(2-methyl-2-oxazoline), poly­(amino acid), and poly­(ethylene glycol) (PEG) are some of the polymers that have been extensively used.^30^

When block copolymers are designed for subsequent polymersome fabrication, one important factor to be considered is the molecular weight ratio of different blocks. The importance of this has previously been demonstrated in the case of PEG-*b*-poly­(alkyl acrylate-*co*-methacrylic acid), in which manipulation of the composition of the ionizable polymer block has been found to alter the performance of the generated product in drug loading and pH-dependent drug release.^31^ Block copolymers with a molecular weight ratio of hydrophilic to hydrophobic blocks of 1:1 are, in general, thought to self-assemble into micelles, whereas those with a ratio of 1:3 tend to form polymersomes.^32,33^ This, however, is only a general trend, and various other factors (such as the packing parameter and the volume fraction of each polymer block) could play a role.^34^ For this, experimentation is often needed to determine the optimal molecular weight ratio of different blocks for a particular block copolymer to form polymersomes.

In addition, currently most of the block copolymers designed for polymersome generation are electrically neutral. Incorporating charged blocks into a block copolymer is, however, one strategy to enhance the functionality of polymersomes through electrostatic interactions. The possibility of generating charged polymersomes has been demonstrated by one study, in which carboxyl groups [whose acid dissociation constant often lies in a range of 3–5, although its actual value could be affected by various factors (ranging from the temperature of the surrounding medium to the type of functional groups copresent in the same chemical entity)^35–39^ ] have been incorporated into PEG–poly­(caprolactone-*graft*-trimethylene carbonate) [PEG-p­(CL-*g*-TMC)] amphiphilic block copolymers.^40^ The rationale of this structural design is based on the understanding that a block copolymer, and the polymersomes thereof, preferably shows high stability at gastric pH (1.5–2) and must be able to disassemble at intestinal pH (6–7.4) if it is to be used as an oral drug carrier.^40^ The polymersomes generated from the copolymers have been found to remain intact at a pH of 5.0 or below, but when the pH of the surrounding medium has been increased to 6.5, deprotonation of the carboxyl groups has occurred, leading to a remarkable increase in the hydrodynamic radius and polydispersity.^40^ Such changes have led to pH-dependent alterations in the mean-square displacement and diffusion coefficient exhibited by the polymersomes.^40^ In fact, over the years, charged polymersomes have already been adopted to achieve better control of the colloidal stability in different media^41^ and to attain on-demand drug release.^42^ Recently, the fabrication of charged polymersomes has been facilitated by advances in microfluidic technologies, with which polymersomes have been successfully generated from poly­(acrylic acid)-*block*-polystyrene (PAA-*b*-PS) by using a flow approach.^43^ The device for continuous-flow polymersome formation enables not only optimization of the self-assembly conditions but also in-line dialysis for the removal of organic solvents.

## Strategies to Generate Polymersomes for Oral Drug Administration

Amphiphilic block copolymers can undergo self-assembly in an aqueous environment to form nanostructures. Such a process is driven predominantly by the tendency of the block copolymers to attain the lowest total free energy of the system (Δ*G* < 0).^44,45^ This is achieved by minimizing, at the expense of the entropy of the single chains, the enthalpy gain caused by hydrophobe–water interactions. The preferentially adopted morphology of the generated self-assembled nanostructures can be predicted by using a dimensionless “packing parameter” (denoted as *p*), which can be calculated by using eq 1:

1 $$p=\frac{v}{a_{0}l}$$

where *v* is the volume of the hydrophobic chains, *a*_0_ is the contact area of the headgroup, and *l* is the length of the hydrophobic tail. In general, when *p* is less than ^1^/_3_, the formation of spherical micelles is favored during the self-assembly process. The micelles are expected to adopt a cylindrical shape when *p* is between ^1^/_3_ and ^1^/_2_. When *p* is further increased to be between ^1^/_2_ and 1, the formation of polymersomes is favored (Figure 1).

**Figure 1:**
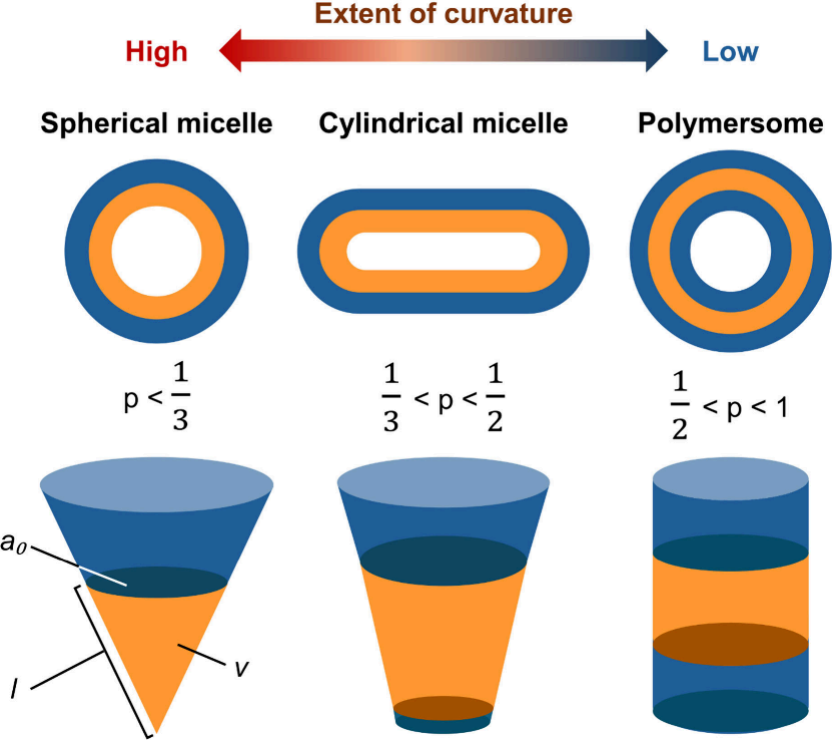
Schematic diagram illustrating the different morphologies of self-assembled structures formed by amphiphilic block copolymers.

Encapsulation of drugs within polymersomes can be achieved in two ways (Figure 2). The first method involves generating polymersomes, followed by electroporation and extrusion to load drug molecules inside.^46^ This method offers flexibility in selecting drug molecules to load after the self-assembly process but is limited to hydrophilic drugs and requires multiple stages. The second method involves mixing drug molecules with amphiphilic block copolymers, allowing the drug to be encapsulated during polymersome formation.^46^ This single-step method enables the loading of both hydrophobic and hydrophilic drugs and is more commonly used. One approach to generating drug-loaded polymersomes via this method is solvent evaporation. This approach has previously been adopted to generate polymersomes from FA-P85-PLGA for oral administration of insulin. During polymersome preparation, a tetrahydrofuran solution of FA-P85-PLGA is first added to an aqueous solution of insulin, followed by constant stirring of the resulting emulsion.^11^ Upon evaporation of the organic solvent, the generated insulin-loaded polymersomes are retrieved by centrifugation before dispersion into water for subsequent use.^11^ Apart from evaporation of the organic solvent from an emulsion to generate polymersomes, some polymersomes could be produced and retrieved by taking advantage of the variations in solubility in different solvents. The use of this method can be exemplified in a recent study,^47^ in which a nanogel–polymersome system [consisting of chitosan diacetate (CDA), methoxypoly­(ethylene glycol)-*b*-poly­(lactide) (MPP), and d-α-tocopherylpoly­(ethylene glycol) succinate (TPGS)] with permeation–glycoprotein inhibition capability has been developed for codelivery of oxaliplatin and rapamycin for chemotherapy.^47^ The polymersomes are generated via solvent switch, in which a dimethyl sulfoxide (DMSO) solution containing MPP and the two drugs is added dropwise to an aqueous solution of TPGS, followed by constant stirring and subsequent dialysis against deionized water.^47^ The generated polymersomes (namely, TMOR) are then modified by nanoparticles generated from CDA, forming TMOR-CDAN, to prolong the residence time (and to prevent degradation) of the loaded drugs in the gastrointestinal tract.^47^

**Figure 2:**
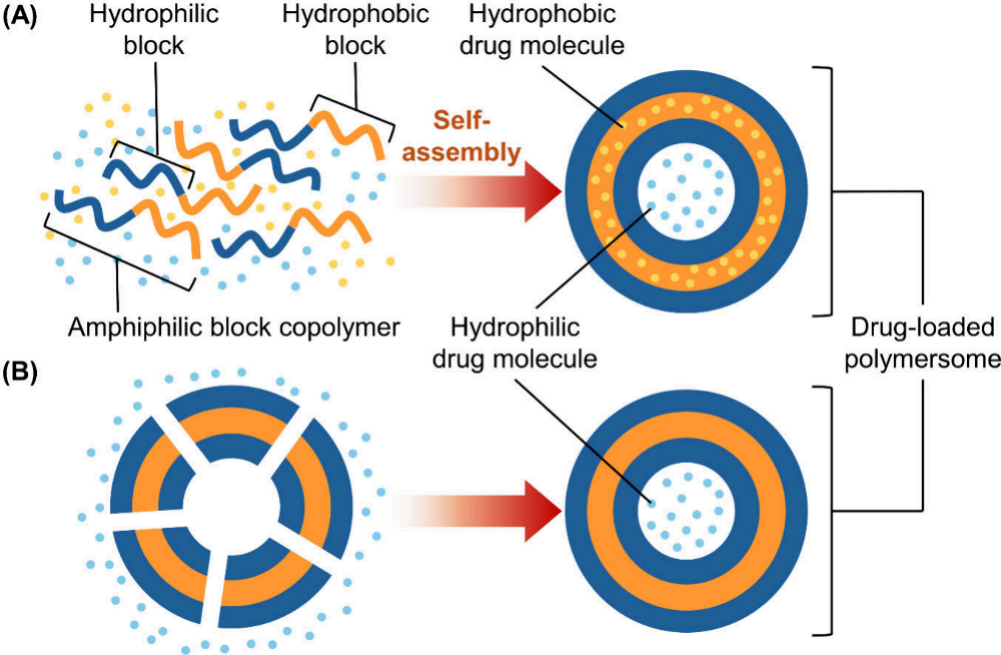
Schematic diagram depicting the formation of drug-loaded polymersomes, which can be achieved by (A) mixing drug molecules with the amphiphilic block copolymer during the self-assembly process or (B) loading drug molecules into preformed polymersomes after self-assembly.

Apart from the methods mentioned above, polymersome-based oral drug carriers can be prepared by thin-film rehydration,^48^ sonication,^49^ and direct dissolution.^50^ Some of these methods have already been reviewed elsewhere.^51,52^ Recently, the fabrication of polymersomes has benefited from advances in microfluidic technologies. For instance, a Y-shaped microfluidic device with a toroidal mixer generated by both photolithography and soft lithography has been used to mix a DMSO solution of a poly­(vinyl alcohol)–PEG block copolymer with deionized water (Figure 3).^53^ In order to minimize the free energy involved,^54^ the copolymer undergoes self-assembly, generating polymersomes for codelivery of nisin and curcumin.^53^ Although the use of microfluidics in polymersome generation is still not as prevalent as conventional methods (e.g., solvent switch and evaporation), over the last several decades, microfluidics has emerged as a compelling technology enabling the generation of single droplets and multiple droplet arrays with precisely controlled composition and size distribution.^55–58^ Up to now, microfluidic technologies have already been applied to diverse areas, ranging from liposome production^59–62^ to the generation of metal nanoparticles.^63,64^ Their tract record of application in fabricating nanoparticulate drug delivery systems, along with their potential to enable automation miniaturization and their capacity of manipulating fluids at a small length scale, is envisaged to contribute to the increasing use of microfluidic technologies in polymersome fabrication and optimization in the upcoming decade.

**Figure 3:**
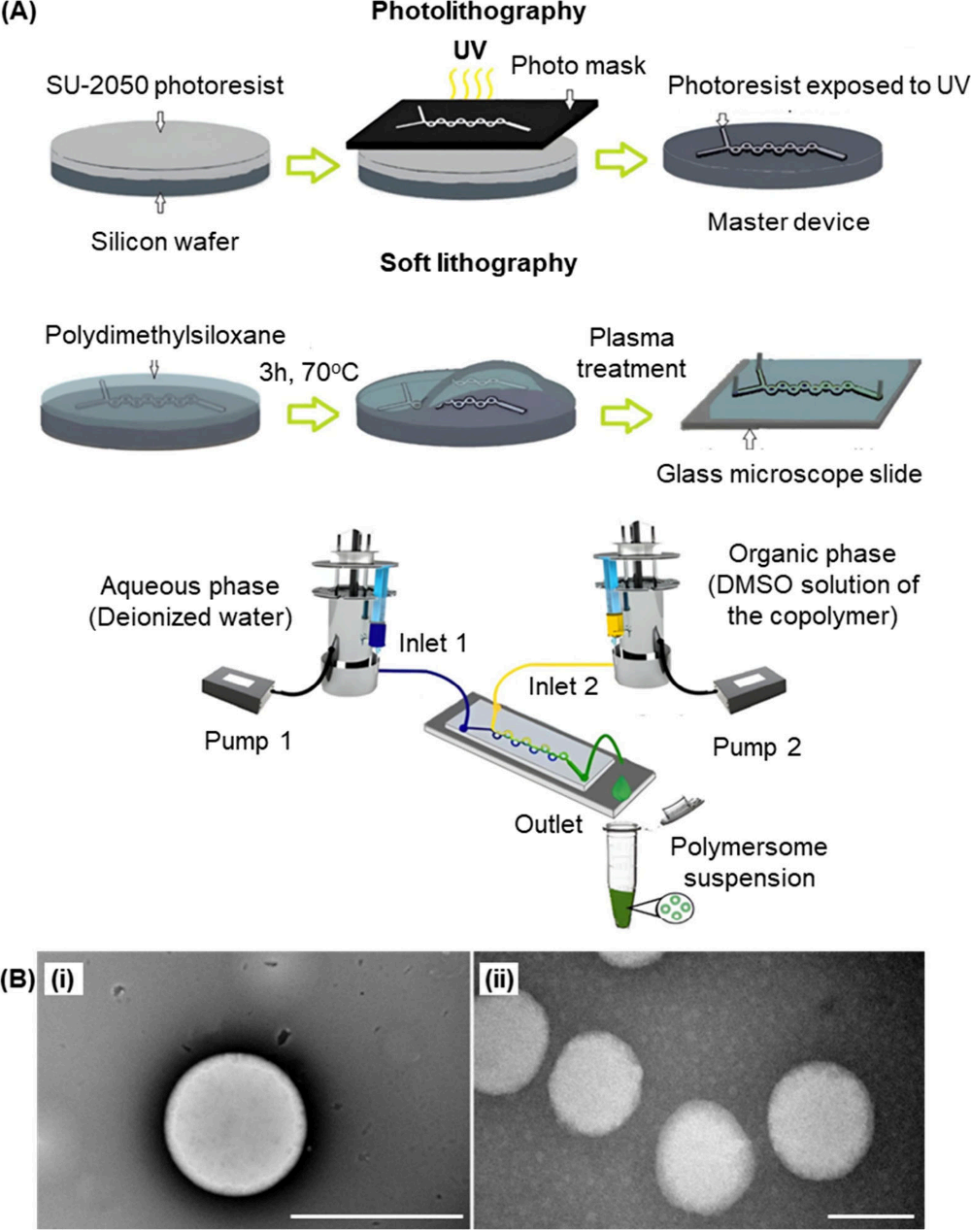
(A) Fabrication of the Y-shaped microfluidic device and the subsequent generation of polymersomes. (B) Transmission electron micrographs of the polymersomes (i) before and (ii) after drug loading. Scale bar = 200 nm. Reproduced with permission from ref 53. Copyright 2024 Elsevier BV.

## Roles and Use of Polymersomes in Oral Drug Administration

The oral route is one of the most preferred routes of drug administration because of not only its ease of operation but also its noninvasiveness and hence high patient compliance. However, it is not without reason that from time to time the parenteral route rather than the oral one is adopted. This is because the efficiency of drugs administered orally is easily impeded by biological and biochemical barriers imposed by the gastrointestinal tract.^65–68^ Examples of biological barriers include the low pH of the gastric environment and the mucus membrane lining the gastrointestinal tract.^69,70^ Biochemical barriers comprise intestinal metabolism (mediated by digestive enzymes), brush-border metabolism (facilitated by enzymes located on the microvilli of enterocytes), and intracellular metabolism (occurring within enterocytes, involving enzymes such as cytochrome P450 and phase II conjugating enzymes).^71,72^ The first-pass effect, referring to the presystemic metabolism of a drug in the intestine and, more significantly, in the liver after absorption and transport via the hepatic portal vein, can also reduce the observed oral bioavailability. In addition, properties of the drug *per se* will significantly influence oral bioavailability. In general, drugs that are classified by the Biopharmaceutical Classification System (BCS) as Class I are ideal for administration via the oral route because these drugs show high solubility and permeability.^73^ On the other hand, the oral bioavailability of BCS Class II, III and IV drugs may not be satisfactory because these drugs exhibit poor solubility and/or poor permeability.^74,75^ Major roles of polymersomes in oral drug administration are therefore either to assist the delivered agent to overcome some of the aforementioned barriers or to modify the properties of the delivered agent to enhance oral bioavailability (Figure 4).

**Figure 4:**
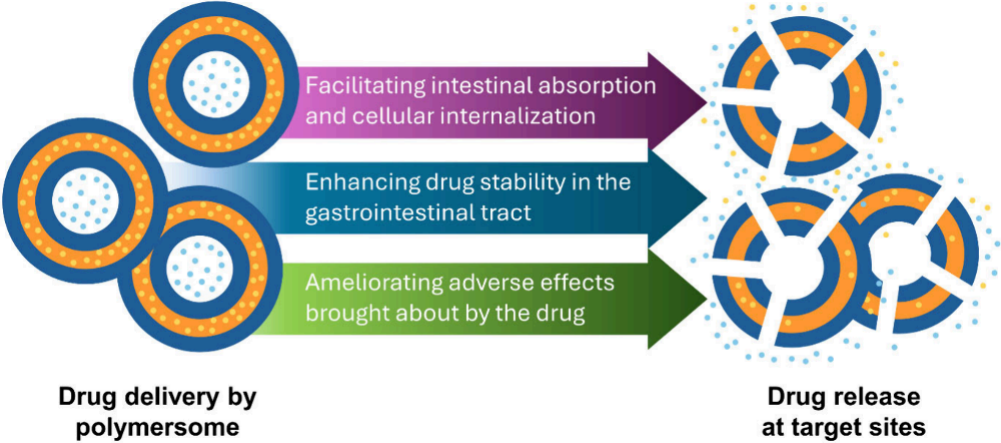
Overview of the major roles played by polymersomes as carriers for oral drug delivery.

### Enhancing Drug Stability in the Gastrointestinal Tract

One major role of polymersome-based oral drug carriers is to enhance the stability of the delivered agent in the gastrointestinal tract. Such technical viability has been demonstrated by the case of rapamycin, which readily undergoes degradation via ring opening under an acidic environment.^76^ The poor stability of this drug makes it highly susceptible to gastric action upon oral administration, leading to low oral bioavailability.^76^ A previous study has demonstrated that more than 90% of free rapamycin has undergone degradation after being incubated at pH 1.2 for 90 min.^47^ Yet, after encapsulation of rapamycin into polymersomes, only 20% of rapamycin has been degraded.^47^ A similar observation of the role of polymersomes in enhancing drug stability has been made on insulin, which is a protein and hence is susceptible to denaturation in the gastric environment. In pepsin-containing simulated gastric fluid (pH 1.2), over 85% of insulin in a free insulin solution has been degraded, whereas only around 35% of insulin encapsulated by FA-P85-PLGA polymersomes has undergone degradation.^11^ Furthermore, in trypsin-containing simulated intestinal fluid (pH 6.8), only less than 15% of insulin in a free insulin solution has been maintained; however, after encapsulation by the polymersomes, the percentage of insulin that has been protected from degradation has reached as high as 76%.^11^ All of these corroborate the role of polymersomes in protecting vulnerable drugs from degradation after oral administration.

Apart from the fragile drugs that are readily degradable, polymersomes can stabilize drugs that are susceptible to metabolism after oral ingestion. This is evidenced by the case of sorafenib, which is known not only to display poor solubility in a wide range of pH values (1.2–7.4)^77,78^ but also to be highly susceptible to first-pass metabolism, thereby having poor oral bioavailability.^79–81^ In an earlier study, polymersomes generated from poly­(butadiene)-*block*-poly­(ethylene oxide) (PB-*b*-PEO) have been used as carriers of sorafenib.^82^ Compared with mice given a sorafenib suspension via the oral route, those orally administered with sorafenib-loaded PB-*b*-PEO polymersomes have been found to have a higher plasma drug concentration and a higher *C*_max_ value. This reveals the success of the polymersomes in protecting the delivered drug from first-pass metabolism upon oral administration. Although the exact mechanism adopted by the polymersomes to achieve this has yet to be fully elucidated, it has been reported that polymeric micelles with appropriate design could redirect the absorption pathway of the encapsulated drug from the portal circulation to the intestinal lymphatic system so as to bypass the first-pass effect in the liver.^83^ Furthermore, polymersomes could be engineered to enhance cellular uptake via mechanisms such as transcytosis,^84^ particularly through M-cells in Peyer’s patches, which may facilitate absorption via routes less exposed to hepatic metabolism.^85^ Together with the fact that polymersomes could provide a protective barrier that shields the encapsulated drug from enzymatic degradation in the gastrointestinal tract, thereby increasing the likelihood that the active drug reaches systemic circulation intact,^2,86^ all of these features may help explain the ability of polymersomes to enhance the oral bioavailability of drugs susceptible to first-pass metabolism.

### Facilitating Intestinal Absorption and Cellular Internalization

Apart from enhancing the oral bioavailability of the delivered drug by improving drug stability, polymersome-based carriers may proactively facilitate intestinal absorption and cellular internalization of the orally administered agent. The viability of using polymersome-based carriers to enhance cellular uptake of the orally administered agent is partially evidenced by poloxamer 401 polymersomes, which have been adopted for oral delivery of proteinaceous agents.^87^ In the epithelial/macrophage coculture model, adalimumab-loaded poloxamer 401 polymersomes have shown the ability to reduce the proinflammatory cytokine level, with the detected concentration of tumor necrosis factor α (TNF-α) being negatively related to the concentration of adalimumab loaded into the polymersomes.^87^ Furthermore, immunoglobulin G (IgG) delivered by the polymersomes has led to 2.7-fold greater intestinal epithelial permeation in Caco-2 cell monolayers compared to unencapsulated IgG.^87^ To elucidate the possible cellular uptake mechanism adopted by polymersome-based carriers, an earlier study has treated Caco-2 cells with chlorpromazine (to disrupt the assembly and disassembly of clathrin), filipin (to disrupt the caveolae structure by binding to cholesterol), and colchicine (to lead to the disassembly of microtubules).^11^ Upon cell treatment, cellular uptake of polymersomes has been found to be inhibited.^11^ This reveals that cellular internalization of the polymersomes could be mediated concomitantly by micropinocytosis, clathrin-mediated endocytosis, and caveolae-mediated endocytosis.

Apart from enhancing cellular internalization, polymersome-based carriers can modulate the absorption profile of the delivered agent in the gastrointestinal tract. This has been demonstrated by the pH-responsive PEG-p­(CL-*g*-TMC) polymersomes developed recently for oral administration of mycophenolate mofetil.^40^ Mycophenolate mofetil is a drug used as an alternative therapy for patients with inflammatory bowel disease unresponsive to conventional treatments.^88^ Its feasibility to be delivered via the oral route has been impeded by its low solubility in the small intestine and its high solubility (and absorption) in the stomach. The aim of delivering the drug using those polymersomes is, therefore, to reduce drug absorption in the stomach and to increase absorption in the small intestine. Upon oral administration of mycophenolate mofetil-loaded polymersomes to male Wistar Han rats that have undergone a 12-h fasting period, a higher amount of the loaded drug has successfully reached the intestinal region even though absorption in the stomach has still been observed.^40^

### Ameliorating Adverse Effects Brought about by the Administered Drug

The technical feasibility of ameliorating adverse effects brought about by the administered drug has been revealed by Wande and co-workers,^47^ who applied nanogel-modified polymersomes to codeliver oxaliplatin and rapamycin for synergistic chemotherapy. In the *in vivo* context, the effectiveness of the polymersomes in mediating chemotherapy via the oral route was confirmed by using the 4T1 subcutaneous carcinoma model, which was established by infiltrating mice with murine mammary carcinoma 4T1 cells into the left axilla.^47^ Compared with using free drugs, reduction of the tumor size was found to be more significant in the group treated with the drug-loaded nanogel-modified polymersomes.^47^ Importantly, the colon length of the treated mice was examined to determine the severity of drug-induced inflammation caused by the treatment.^47^ Compared with those treated with free drugs, those treated with the drug-loaded nanogel-modified polymersomes were shown to undergo less significance of colon shortening.^47^ This reveals that polymersomes have played a role in reducing chemotherapy-induced gastrointestinal toxicity.

This amelioration of adverse effects can be attributed to the ability of polymersomes to offer controlled or sustained drug release, minimizing sudden spikes in the systemic drug concentration that can trigger toxicity. The coencapsulation of drugs also allows for synergistic action at lower doses, potentially reducing the need for high concentrations of each agent and thereby limiting side effects. Apart from these, polymersomes can shield sensitive cell membranes from direct contact with the administered agents to improve the safety profile of those agents. This has been confirmed by an earlier study,^82^ which treated human erythrocytes with a suspension of sorafenib (200 μg/mL) and found that around 9% of the treated cells underwent hemolysis. On the other hand, upon encapsulation by PB-*b*-PEO polymersomes, the percentage of hemolysis was found to be negligible.^82^ Altogether, the role of polymersome-based carriers in mitigating adverse effects of orally administered agents results from their combined ability to modulate drug release, lower the effective dose, and limit cellular exposure to those agents.

## Optimization for Enhanced Performance in Oral Drug Delivery

The performance of polymersomes in oral drug delivery is affected largely by the structure of the amphiphilic block copolymers, as well as the properties of the generated polymersomes. For this, optimization of the delivery efficiency mediated by polymersome-based oral drug carriers is generally conducted in these aspects. In the following section, major strategies to enhance the design and preparation of polymersomes are discussed for oral drug administration.

### Manipulation of the Structural Properties of Block Copolymers

Polymersomes are generated from the self-assembly of amphiphilic block copolymers. Changing the structure of these copolymers leads to an alteration in the self-assembly process and the structure of the generated nanoparticulate systems. This has been demonstrated by the case of the PEG–poly­(*D*,*L*-lactide) (PLA) copolymer. By fixing the molecular weight of PEG at 5 kDa and varying the block length of PLA, the copolymer was found to form micelles when the PLA block had a molecular weight of 5 kDa and transitioned to forming polymersomes as the molecular weight of the PLA block increased to 15 kDa (Figure 5).^5^ This is largely due to the bulkiness of the hydrophobic PLA segment, making it fail to fit in the interior of a micelle and hence forming a bilayer structure instead.^5^ In addition, altering the molecular weight of hydrophobic segments could lead to changes in structural features (particularly the membrane thickness) of the generated polymersomes. Because polymersomes have a structure consisting of an aqueous core, along with a hydrophobic membrane and hydrophilic corona, increasing membrane thickness has been found to facilitate the loading of hydrophobic agents. This has been shown to be feasible in previous studies, in which polymersomes have been used to deliver paclitaxel^89^ and sorafenib.^82^

**Figure 5:**
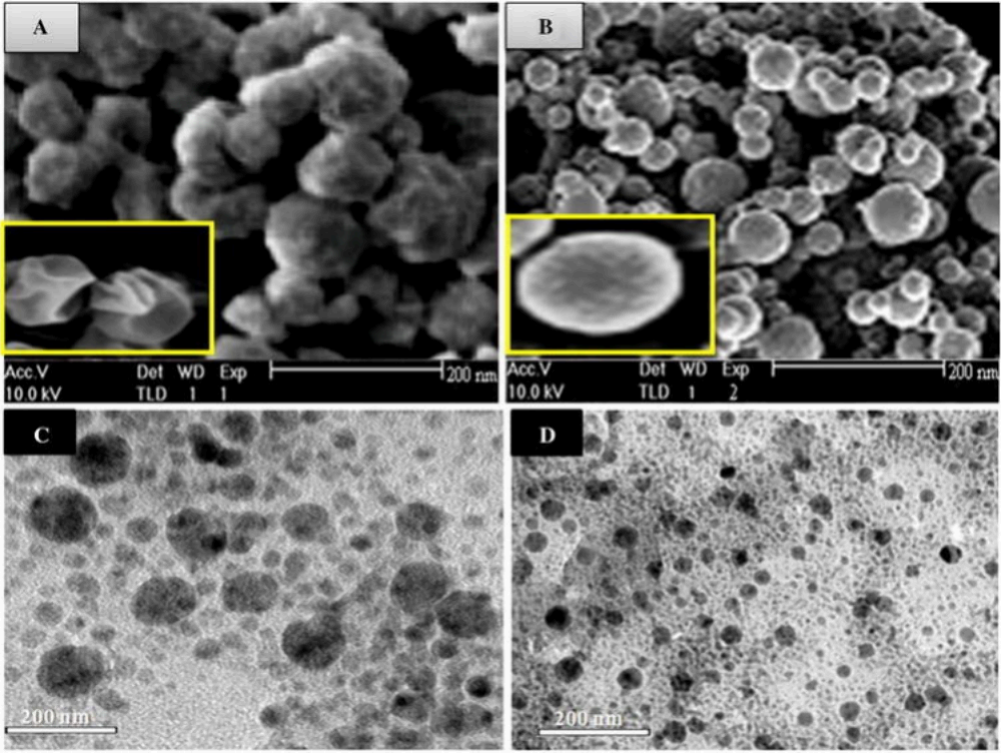
SEM images of (A) polymersomes and (B) micelles. TEM images of doxorubicin-loaded (C) polymersomes and (D) micelles. Reproduced with permission from ref 5. Copyright 2015 Springer Nature.

Furthermore, to enhance the controlled release of an orally administered drug, various functionalities sensitive to the pH, redox conditions, or various physiological factors could be incorporated into a drug delivery system. This approach has been adopted in various types of carriers, ranging from metal–organic frameworks^90^ and composite gels^91,92^ to polymeric nanoparticles.^93,94^ In terms of polymersomes, this approach can be adopted by incorporating the respective functionalities into block copolymers before polymersome fabrication. The possible use of this approach has been partially demonstrated by the case of polymersomes generated from PEG-p­(CL-*g*-TMC), in which carboxyl groups have been added to render the subsequently generated polymersomes pH-responsive, for oral delivery of immunosuppressants.^40^ Release of the loaded drug from the polymersomes has been found to be initiated when the pH of the surrounding medium reaches 6.5 (pH of the duodenum) or 7.5 (pH of the small intestine), with 90% of the loaded drug being released within the first 2 h.^40^ The release profile fits well with the Korsmeyer–Peppas model and follows non-Fickian diffusion.^40^ The success of achieving controlled release in the temporospatial sense can enhance the oral bioavailability of the delivered drug by ensuring its release occuring only after the carrier reaches the desired site of action.

### Optimization of Preparation Conditions

To optimize the performance of polymersome-based oral drug carriers for preclinical and clinical translation, the self-assembly conditions have to be properly controlled during the preparation of polymersomes because they could significantly influence the structure of the generated self-assembled systems. This has been revealed by a recent study, in which PAA-*b*-PS polymersomes have been generated using a flow self-assembly setup.^43^ In the setup, a stream consisting of PAA-*b*-PS in tetrahydrofuran is coflowed with a stream consisting of hydrochloric acid (HCl) (which, on the one hand, can modulate the charged state of the PAA blocks and, on the other hand, can induce the self-assembly of the copolymer due to its nonsolvent nature with respect to PS) (Figure 6). Results showed that changing either the concentration of HCl or the content of tetrahydrofuran could lead to the formation of different self-assembled structures (micelles, polymersomes, and solid particles). In brief, when the concentration of HCl is low, the PAA blocks of PAA-*b*-PS tend to be deprotonated. This results in charge repulsion, leading to the formation of a comparatively high hydrophilic volume fraction, favoring micelle formation. On the other hand, if the concentration of HCl is too high, the PAA blocks of PAA-*b*-PS will be fully protonated. This results in an increase in the hydrophobic volume fraction, favoring the formation of particles deficient of apparent membrane or internal structures. Here it is worth noting that the optimal conditions of polymersome preparation may vary not only from one block copolymer to another but also from one application to another. For this, the preparation conditions should be optimized based on the characteristics of the specific amphiphilic block copolymer and the need for the specific application. This has been partly evidenced in the case of PB-*b*-PEO, in which the critical aggregate concentration for polymersome formation has been found to be affected by the molecular weight.^82^ The optimal concentration of the copolymer for polymersome preparation, therefore, has to be determined in a case-by-case manner.

**Figure 6:**
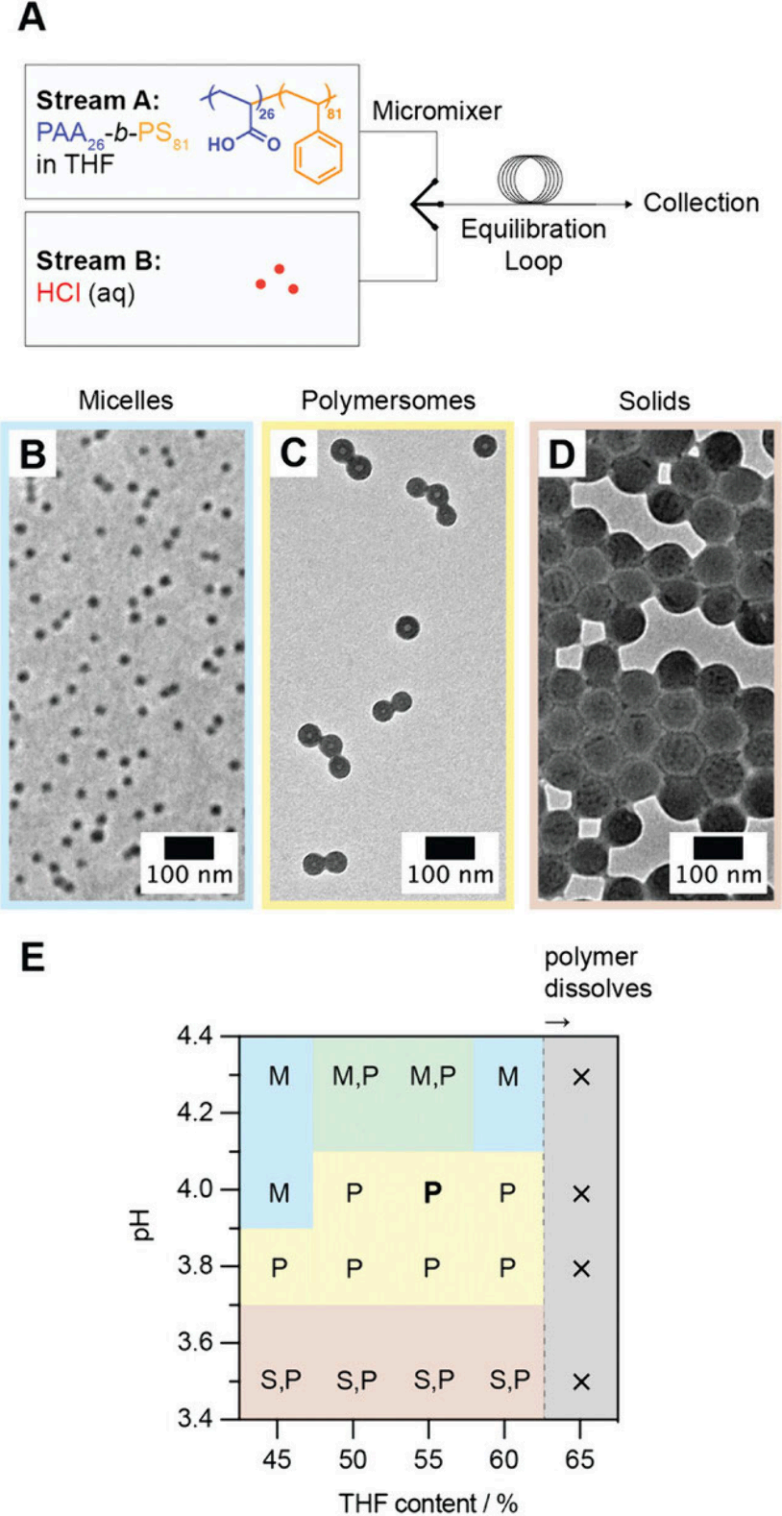
(A) Schematic diagram depicting the flow self-assembly setup. (B–D) TEM micrographs of self-assembled structures obtained from PAA-*b*-PS. (E) Phase diagram depicting changes in the self-assembled structures under different combinations of pH and tetrahydrofuran content. In the figure, M, P, and S denote micelles, polymersomes, and solid particles, respectively. Reproduced with permission from ref 43. Copyright 2024 John Wiley & Sons, Inc.

### Refinement of the Physicochemical Properties of Polymersomes

Once polymersomes are generated, their physicochemical features (ranging from size and surface properties to morphology) could remarkably influence their performance in oral drug administration. From a physiological perspective, the mucus layer, with its mesh-like network and brush-like architecture, functions as a size-selective barrier that restricts the movement of large molecules.^95,96^ Particles generally need to be smaller than 200 nm in order to effectively penetrate the mucus layer.^97^ As far as the size of polymersomes is concerned, it is worth noting that the size of plain polymersomes may not effectively predict the pharmacokinetic profile exhibited by the drug-loaded polymersomes upon oral injection. This is due to the fact that the size of polymersomes could be changed upon drug loading. The possibility of this has been demonstrated by the case of PEG-p­(CL-*g*-TMC) polymersomes, whose hydrodynamic diameter increases from 90.8 ± 1.2 to 106.7 ± 1.9 nm upon drug encapsulation.^40^ In addition, changing the amount of a loaded drug could significantly alter the hydrodynamic diameter of generated polymersomes, leading to changes in the pharmacokinetic profile. This has been reported by Wande and co-workers,^47^ who found that, by changing the amount of oxaliplatin loaded into polymersomes from 1 to 10 mg, the size of the generated polymersomes changed from over 300 nm to around 155 nm and back to over 300 nm again. For this, characterizing the size of polymersomes should be done after the drug-loading process, with the identity and amount of the loaded drug being known at the time of size determination.

Not only the size but also the ζ potential of a carrier can influence the efficiency of oral drug delivery. Negatively charged and neutral particles, in general, penetrate the mucus layer more easily.^98^ In contrast, positively charged particles exhibit lower mobility in mucus but greater cellular uptake via endocytosis than their negatively charged counterparts.^99,100^ Controlling the ζ potential is, therefore, crucial in the design of polymersomes. Yet, it is important to note that the ζ potential of polymersomes may change during the process of drug loading. This has been hinted at by the case of the self-assembled carrier formed by the PLGA–PEG–PLGA copolymer. While increasing the concentration of loaded US597 from 3 to 30 mg/mL has been found not to have a significant effect on the encapsulation efficiency and loading efficiency,^101^ an increase in the ζ potential from 5.76 ± 1.1 to 10.65 ± 1.5 mV has been observed. Such a change may be due to the coating of the self-assembled carrier with US597.^101^ During the drug-loading process, while the hydrophobic PLGA blocks in the core interact with the lipophilic rigid triterpenoid ring structure of US597, the hydrophilic PEG blocks on the surface of the self-assembled structure also interact with the polar NH_2_ group of the drug. Such polymer–drug interactions lead to changes in the ζ potential of the drug-loaded carrier. Here it is worth noting that when the drug to be delivered possesses both hydrophilic and lipophilic groups, care should be taken in carrier design to avoid an initial burst release. Taking the US597-loaded carrier mentioned above as an example, due to the rapid dissociation of surface-bounded drug molecules, a significant initial burst release has been observed.^101^ The release profile turns out to be sustained and steady only after 40 h, after which the release of drug molecules entrapped inside the self-assembled structure becomes dominant.^101^

Apart from optimizing the size and ζ potential, surface modification can help enhance the efficiency of polymersomes in oral drug administration. This has been shown by the case of PLGA–P85–PLGA polymersomes. Upon oral administration of the insulin-loaded polymersomes to fasting diabetes rats, a blood glucose depression of 25.3% at 2 h and 43.7% at 4 h was observed.^11^ However, upon incorporation of folate onto the surface of the insulin-loaded polymersomes, the blood glucose depression achieved was increased to 36.8% at 2 h and 59.3% at 4 h.^11^ In addition, compared with the AUC value of the insulin-loaded PLGA–P85–PLGA polymersomes (211 ± 19.7 μ IU h/mL), that of the folate-incorporated ones was reported to be 1.27-fold higher,^11^ leading to a substantially higher plasma insulin concentration (27.6 ± 3.67 μ IU/mL for the insulin-loaded PLGA–P85–PLGA polymersomes vs. 35.8 ± 5.27 μ IU/mL for the folate-incorporated ones) 6 h after oral administration to diabetes rats.^11^ Besides the incorporation of ligands, the polymersome surface could be modified morphologically. The technical feasibility of this has been demonstrated by Thomas and co-workers,^102^ who adopted nucleobase pairing to direct the formation and lengthening of nodes on the outer surface of polymersomes. By adding a short diblock copolymer possessing complementary thymine side chains onto the surface of the polymersomes, an increase in steric crowding at the hydrophilic/hydrophobic interface resulted.^102^ Such steric crowding subsequently initiated node formation and elongation (Figure 7).^102^ Once the morphology of the polymersome surface can be fine-tuned, it is anticipated that the pharmacokinetic profile of polymersomes could be better tailored to meet different needs of oral drug administration.

**Figure 7:**
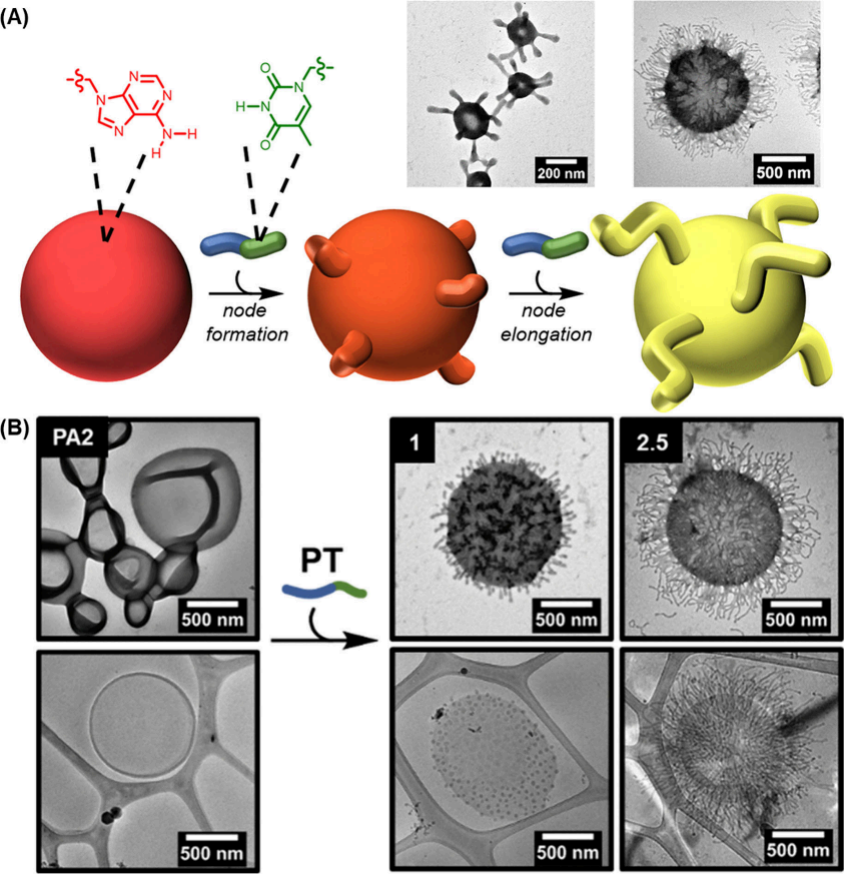
(A) Schematic diagram showing the process of node formation and node elongation on the surface of polymersomes. The process is achieved by adding a diblock copolymer, namely PT, which contains complementary thymine side chains and is synthesized via aqueous reversible addition–fragmentation chain-transfer polymerization-induced self-assembly, onto the polymersome membrane. (B) Dry-state and cryo-TEM images depicting the formation and lengthening of nodes on the surface of the polymersomes. Reproduced with permission from ref 102. Copyright 2024 Royal Society of Chemistry.

Last but not least, the shape of polymersomes could also be manipulated. Changing the shape of polymersomes can not only alter drug release kinetics by modifying both the surface-to-volume ratio and aspect ratio of the system^103–105^ but can also influence the polymersomes' interactions with intestinal cell surfaces and the degree of mucosal entrapment they experience.^105,106^ The shape of polymersomes is, therefore, an important consideration in the design of carriers for oral drug delivery.^107^ It can be manipulated by altering the structure of a block copolymer. This has been shown by the polymersomes generated by using a block copolymer consisting of a hydrophilic PEG block and a hydrophobic poly­(trimethylene carbonate–azobenzene) [P­(TMC-AZO)] block.^108^ The degree of polymerization of the P­(TMC-AZO) block was found to determine the morphology of the self-assembled structures. By having the number of monomers in the P­(TMC-AZO) block to be 12, small micelles with a diameter of around 20 nm formed upon self-assembly of the copolymer.^108^ Increasing the number of monomers in the block to 20–25 resulted in larger micelles that were interconnected.^108^ A further increase in the length of the P­(TMC-AZO) block led to the formation of ellipsoid-like vesicular nanostructures.^108^ When the number of monomers in the P­(TMC-AZO) block reached 45, tubular polymersomes were obtained.^108^ The generated tubular polymersomes exhibited photoresponsive behavior upon UV/vis light irradiation and transformed into linear micelles upon light stimulation.^108^ Although the polymersomes have not yet been tested for oral drug delivery, the technical feasibility of manipulating the morphological features of polymersome-based oral drug carriers has been corroborated.

## Opportunities and Challenges

As far as oral drug administration is concerned, polymersomes have been used only as discrete nanoparticulate systems for drug delivery in the literature. In fact, polymersomes have the potential to serve as colloidal building blocks to generate higher-order clustered structures. This can be achieved by using not only DNA base-pairing interactions to bind polymersomes with other colloidal components^109–111^ but also electrostatic interactions. The feasibility of the latter has been demonstrated by a recent study,^112^ in which positively charged polymersomes were used as core particles to which negatively charged micelles electrostatically attached as satellite particles (Figure 8). The positive charge of the polymersomes and the negative charge of the micelles come from the presence of PAA and quaternized poly­(4-vinylpyridine), respectively, in their structures. Such an approach of clustering enables the buildup of higher-order structures from polymersomes regardless of the degree of fluidity of the polymersome membrane. Examining the impact of variations in the hierarchical structures generated by polymersomes on the pharmacokinetic profile of the loaded drug upon oral administration will potentially increase our understanding of carrier design and be one of the promising directions for future research.

**Figure 8:**
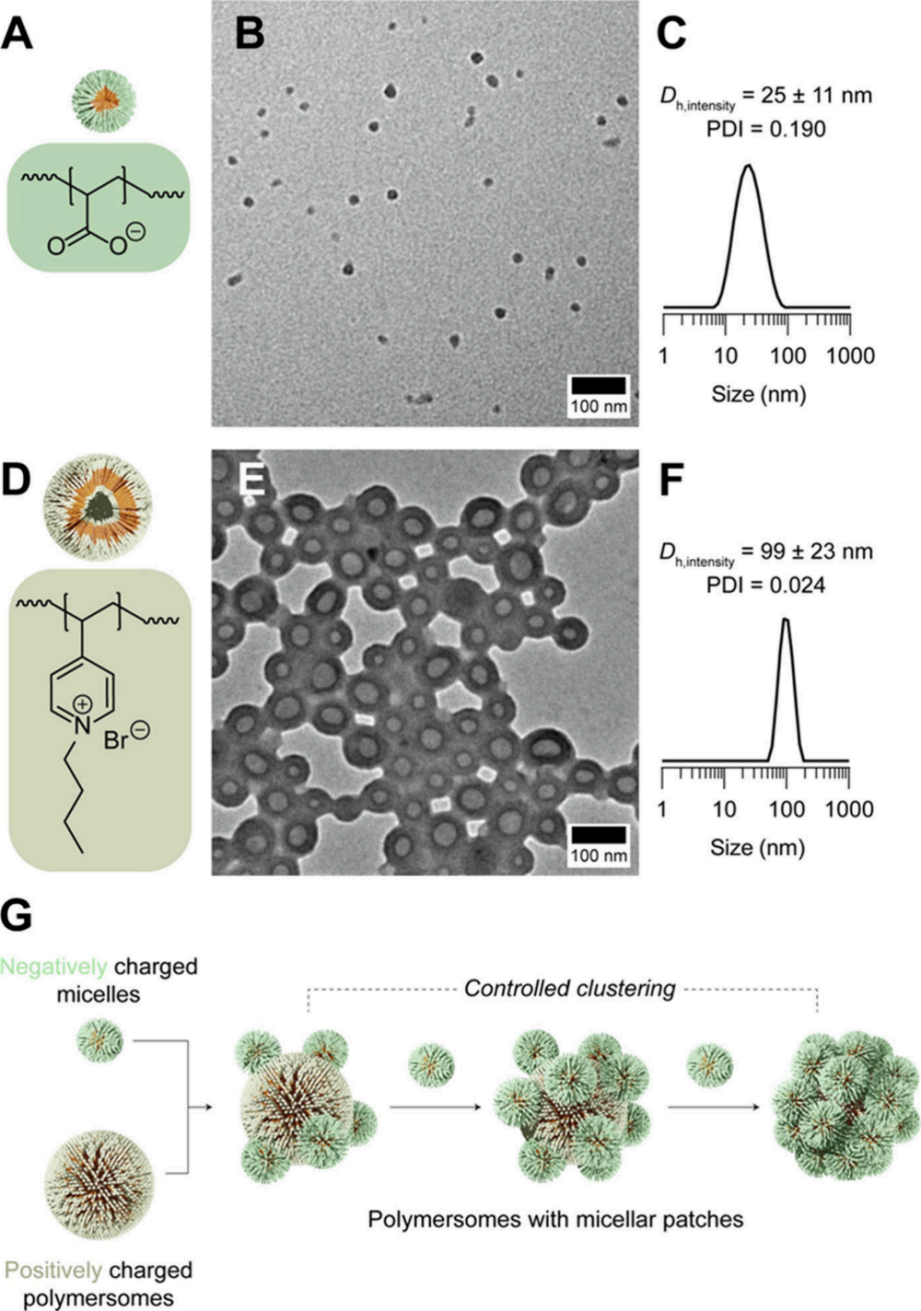
(A) Chemical structure of PAA, which imparts negative charges to the surface of micelles. (B) TEM micrograph and (C) intensity-averaged dynamic light scattering data of the negatively charged micelles. (D) Chemical structure of quaternized poly­(4-vinylpyridine), which imparts positive charges to the surface of polymersomes. (E) TEM micrograph and (F) intensity-averaged dynamic light scattering data of the positively charged polymersomes. (G) Schematic diagram depicting the formation of polymersomes with micellar patches. Reproduced with permission from ref 112. Copyright 2024 Elsevier BV.

Modification of polymersomes with other types of nanoparticulate systems is another area that is worth paying attention to in future research because this can enhance modulation of the functionality of polymersomes in oral drug delivery. For example, by incorporating a near-infrared fluorescent dye and a paramagnetic probe [viz., gadolinium­(III) cations] into polymersomes generated from poly­(acrylic acid-*co*-distearin acrylate), the polymersomes show potential to be used as a diagnostic tool for magnetic resonance imaging and near-infrared imaging.^113^ More recently, a study incorporated polymeric nanoparticles into polymersomes and successfully enhanced the delivery efficiency to the intestinal region.^47^ When plain polymersomes were used at pH 1.2, 84% of loaded oxaliplatin and 36% of loaded rapamycin were released within the first 2 h; however, after modifying the polymersomes with polymeric nanoparticles, less than 12% of the loaded drugs were released under simulated gastric conditions.^47^ This implies that a large percentage of the loaded drugs could be delivered to the intestines and corroborates the feasibility of modulating the oral drug delivery performance by merely incorporating external nanoparticles into the polymersome-based carrier.

Here, it is worth mentioning that while the effect of polymersomes in increasing the percentage of orally administered drugs to reach the intestinal region has been widely demonstrated in the literature, possible retention of polymersomes in the stomach due to the mucoadhesive properties of the block copolymer should not be overlooked and it may reduce, rather than increase, the overall oral bioavailability of the delivered agent.^40^ This concern has been raised by Tollemeto and co-workers,^40^ who used a technique based on quartz crystal microbalance with dissipation (QCM-D) to confirm mucosal retention of polymersomes. Characterizing mucosal retention has been technically challenging outside the body, but light has been shed recently by Hearnden and co-workers,^114^ who first seeded primary oral keratinocytes and oral fibroblasts onto de-epithelialized dermis (DED), followed by raising the cell-attached DED to an air–liquid interface to facilitate the occurrence of epithelial stratification.^114^ With the use of confocal laser scanning microscopy (CLSM), the penetrating capacity of rhodamine-labeled polymersomes in the 3D tissue-engineered oral mucosa was successfully determined.^114^ Such a technique makes *ex vivo* evaluation of the penetration and retention of polymersomes in a mucosal membrane technically feasible. A similar approach has also been reported for investigating penetration of many other nanostructures administered via diverse routes of administration.^115–117^ The penetration and retention of polymersomes upon oral administration is worth exploring in upcoming studies when their performance in oral drug delivery is examined.^118^

Finally, although numerous polymersomes have been developed and tested since the turn of the last century, the transition from laboratory research to clinical trials has yet to be successfully achieved. One major barrier to clinical development lies in the complexity of polymersome formulations and the lack of scalable manufacturing methods. The synthesis of block copolymers often requires multiple steps,^119^ making it difficult to achieve batch-to-batch consistency at an industrial scale. The lack of manufacturing standardization is another factor posing challenges for commercial viability during the clinical translation of polymersome research. To overcome this issue, future research should focus on developing simpler, aqueous-based, or solvent-free synthetic routes that are scalable and reproducible. Furthermore, although many studies have reported promising *in vitro* results and positive outcomes in small animal models,^120–124^ few have extended these findings to large animal models or clinically relevant disease systems, not to mention elucidating the long-term pharmacokinetics of polymersomes. Given that some polymersomes are constructed from nonbiodegradable or partially degradable polymers such as poly­(ethylene oxide)-*b*-poly­(butadiene)^125–127^ or poly­(styrene)-based blocks,^128–130^ they may accumulate in tissues and induce long-term toxicity. Exploring the use of fully biodegradable and biocompatible polymers in polymersome design, as well as incorporating stimuli-responsive linkages that degrade under physiological conditions, would be some of the promising directions for future research.

Regulatory challenges also present an obstacle to clinical translation. Although polymersomes have been studied for decades, currently, there are no approved products or established regulatory precedents that could serve as benchmarks. This creates uncertainty around the requirements for preclinical data and safety assessments. Owing to unclear regulatory pathways and uncertain market returns, pharmaceutical companies are generally hesitant to invest in polymersome-based technologies. This hesitancy further constrains the clinical development of polymersome-mediated oral drug delivery. Addressing this problem will require a proactive engagement with regulatory agencies to define acceptable parameters for clinical progression. Collaborative efforts among researchers, industry partners, and regulatory bodies will be essential to creating a supportive framework for the clinical evaluation of polymersomes.

## Concluding Remarks

Polymersomes, as self-assembled nanostructures generated from amphiphilic block copolymers, exhibit high biocompatibility, excellent stability, and remarkable property tunability, making them promising candidates for drug administration, including oral drug delivery. As detailed in the sections above, the application potential of polymersomes as oral drug carriers has been supported in the literature. The increase in the understanding of self-assembly kinetics, as well as of the factors influencing the pharmacokinetic profiles of orally administered agents, has facilitated the performance enhancement of polymersome-based oral drug carriers. With ongoing advances in artificial intelligence and molecular modeling, not only the elucidation of possible interactions (in forms of fusion and fission) among polymersomes upon oral ingestion but also the possibility of merging multiple block copolymers in polymersome fabrication are expected to be streamlined in the upcoming decade through computational simulations. These simulations, including coarse-grained simulation (in which a cluster of atoms are combined into one interaction particle, enabling modeling of complex polymeric systems), allow the molecular details underlying the mechanism and behavior of polymersome formation to be studied in a way that can hardly be achieved experimentally. Along with the increasing sophistication of the design of polymersomes, the role that polymersomes play in oral drug administration is envisaged to be increasingly prominent in the coming years.

Despite the promising potential of polymersomes in oral drug delivery, further research is required to fine-tune the hydrophilic/hydrophobic block ratio in amphiphilic block copolymers for polymersome fabrication to optimize drug release kinetics. Achieving controlled release at precise locations in the gastrointestinal tract is essential, with stimuli-responsive block copolymers offering a potential solution. However, consistent and predictable release profiles across different physiological conditions must still be established. Additionally, the ability to scale up production from the laboratory to industrial scale is a critical challenge. Current methods of polymersome fabrication struggle with maintaining uniformity in the polymersome size, thereby limiting commercial viability. Microfluidic techniques may offer a solution to optimizing production, although further improvements are needed for industrial-scale reproducibility. To date, the impact of surface modifications on the targeting efficiency and cellular uptake of polymersomes in the gastrointestinal tract has yet to be fully elucidated. Mucosal interaction is another concern as unwanted mucoadhesion could hinder drug delivery to the small intestine. Finally, potential immunogenicity or long-term safety implications from repeated oral administration of polymersomes need thorough investigation. These challenges must be addressed before widespread clinical application of polymersomes in oral drug delivery can be achieved.
